# About a rare cause of calf pain in an athlete: the popliteal artery entrapment syndrome (a case report)

**DOI:** 10.11604/pamj.2021.38.14.27415

**Published:** 2021-01-06

**Authors:** Ameni Ammar, Mahmoud Smida, Mohamed Samir Daghfous

**Affiliations:** 1 Traumatology Department, KASSAB Institute, Manouba, Tunisia

**Keywords:** Popliteal artery entrapment syndrome, asymptomatic diseases, anterior cruciate ligament injuries, case report

## Abstract

Claudication of the young patient is a very rare symptom for orthopaedic surgeons and it is often overlooked. We report a rare case of popliteal artery entrapment syndrome (PAES), discovered during a vascular claudication following post-traumatic anterior instability of the knee. The diagnosis was confirmed by CT angiography which showed a PAES, with a pathway in the inter-condylar notch. The patient had a releasing of the trapped vessel by myomectomy, with disappearance of vascular symptoms six months later. Through this case, we wanted to draw the attention of orthopaedic surgeons to the fact that the PAES can be asymptomatic. Its symptomatology can be triggered by a traumatic instability of the knee. Its presence represents a risk of lesion of the popliteal artery during arthroscopic ACL reconstruction. Therefore, it is important to think about this disease if a calf pain occurring after a ligament injury of the knee.

## Introduction

Subsequent pain after a central ligament injury of the knee is usually attributed to meniscal or osteochondral damage inherent in trauma or secondary to knee instability. It has not been reported that vascular calf pain can be triggered by knee ligament trauma. Claudication of the young patient is a very rare symptom for orthopaedic surgeons and it is often overlooked. We report a rare case of popliteal artery entrapment syndrome (PAES), discovered during a vascular claudication following post-traumatic anterior instability of the knee. PAES is a rare anatomical anomaly characterized by an anomalous relationship between the popliteal artery and the myofascial structures of the popliteal fossa [1]. It can lead to popliteal artery thrombosis, stenosis, distal arterial thromboembolism or arterial aneurysm [2]. PAES is poorly known by orthopaedic surgeons. Its presence represents a risk of lesion of the popliteal artery during arthroscopic ACL reconstruction. Through this case, we wanted to draw the attention of orthopaedic surgeons to this disease.

## Patient and observation

**Figure 1:**
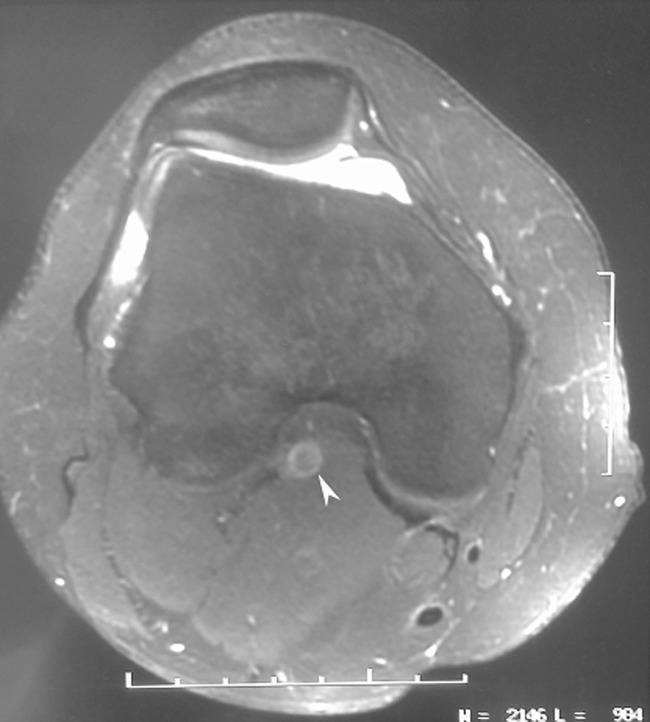
IRM showing the popliteal artery laminated and trapped between the gastrocnemius muscle and the medial condyle

A 29-year-old judo player, having no pathological history consulted for instability of the left knee following a sports accident two months ago. The patient reported that, since traumatism, he had intermittent claudication occurring after walking and during re-education sessions, especially during the reinforcement muscle exercises. Examination of the knee showed anterior laxity. There was no meniscal syndrome and the peripheral pulses were present. MRI of the knee showed anterior cruciate ligament (ACL) rupture. The popliteal artery was laminated and trapped between the gastrocnemius muscle and the medial condyle (Figure 1).

**Figure 2:**
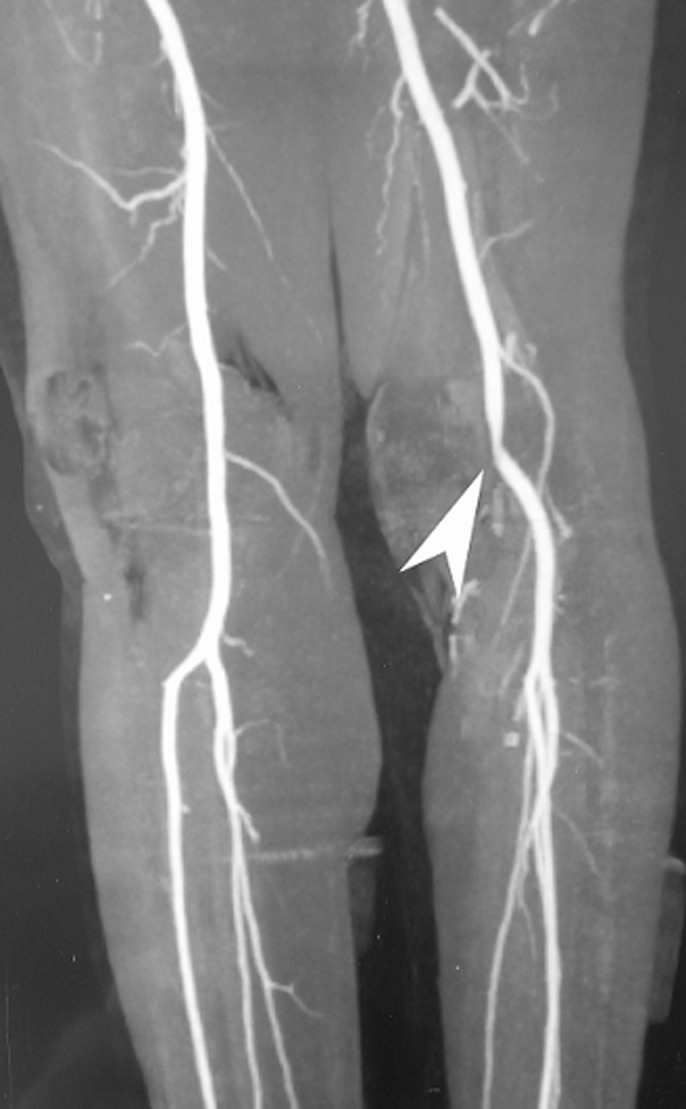
CT Angiography showing a partial occlusion of the popliteal artery due to the entrapment syndrome (PAES)

CT angiography showed a partial occlusion of the popliteal artery due to a popliteal artery entrapment syndrome (PAES) (Figure 2), with a pathway in the inter-condylar notch, flush with the femoral footprint of the native ACL. This made it difficult to drill the femoral tunnel for ACL reconstruction. The patient was warned that this situation represents a risk of popliteal artery injury during arthroscopic ACL reconstruction. The patient was operated on by a vascular surgeon: he had a releasing of the trapped vessel by myomectomy. ACL reconstruction was postponed. After six months, the patient had no vascular claudication.

## Discussion

Popliteal artery entrapment syndrome (PAES) is an uncommon anatomical anomaly and poorly known by orthopaedic surgeons. It is characterized by various anomalous anatomic relationships between myofascial structures and arteries in the popliteal fossa, resulting in extrinsic arterial compression [1,2]. The PAES can be congenital or acquired [1,2]. It mostly occurs in young adults with well-developed muscles [3,4]. More rarely, it has been described in children presenting with acute lower limb ischaemia caused by PAES [5]. The PAES cases were classified according to the Love and Whelan classification modified by Rich [6]: type I: the popliteal artery has an aberrant medial course around the medial head of gastrocnemius, which has a normal insertion above the femoral condyle; type II: the medial head of gastrocnemius is inserted more laterally on the distal femur, with medial displacement of popliteal artery; type III: an aberrant accessory slip of medial head of gastrocnemius slings around and surrounds the normally positioned popliteal artery; type IV: the popliteal artery is located deep in the popliteal fossa and entrapped by the popliteus muscle or fibrous bands; type V: any form of entrapment that involves both popliteal artery and popliteal vein.

The most common symptoms associated with PAES are intermittent claudication and pain in the feet and calves after exercise. In more severe cases of PAES, the symptoms are caused by complications that include thrombosis, arterial aneurysm, arterial stenosis or distal embolism. On physical examination, there may be decreased or absent pulses during forced dorsal foot flexion or signs of decreased perfusion such as pallor, a lower limb thermal gradient and limb cyanosis in acute cases [7]. Imaging plays a central role in diagnosing PAES. Ultrasound may show the arterial displacement and the presence of a fibrous band between the popliteal artery and vein. Popliteal stenosis, post-stenotic dilation and thrombus may be seen in the case of long-standing disease. Diagnosis of functional PAES relies upon evidence of arterial diameter reduction and increased maximum systolic velocity at the site of compression upon dynamic testing. Ultrasound is the modality of choice for this functional testing, given its inherent adaptability for dynamic imaging [8].

Magnetic resonance imaging (MRI) is the best method for evaluation of the anatomy of the popliteal fossa as this imaging method may accurately show the structures surrounding the popliteal artery in this region [8]. Angiography detects compression and occlusion of the popliteal artery and may show an abnormal route of the artery. If no changes are seen on vascular imaging, the patient may be asked to perform plantar dorsiflexion until becoming symptomatic. Repeated angiography may detect so abnormalities [8]. The use of MRI with arteriography has been reported to provide the most accurate diagnostic approach for PAES [7]. MR and CT angiography offer precise analysis of the medial deviation of the vessel by an abnormal slip of the medial or lateral head of the gastrocnemius muscle or entrapment of the popliteal artery by a fibrous band [5]. PAES is a progressive condition, for which early diagnosis is warranted to prevent serious complications, including post-stenotic aneurysms and distal embolization, with risk of subsequent ischemia [7]. PAES can be bilateral and therefore screening of the contralateral knee is warranted in case of PAES [9].

The treatment can be medical or surgical. The purpose of the surgical procedure is to restore the abnormal relationship between the artery and the internal head of the gastrocnemius. Treatment consists of releasing the trapped vessel by myomectomy. The presence of vascular complications such as stenosis, aneurysm or occlusion warrants additional vascular reconstruction by endarterectomy or bypass grafting. Endovascular treatments are not effective in PAES and are associated with high rates of re-occlusion unless the underlying cause of entrapment is treated [1]. For bilateral PAES it is recommended that both sides are treated, although asymptomatic, to avoid later acute ischaemia [5].

## Conclusion

Popliteal artery entrapment syndrome (PAES) is an uncommon anatomical anomaly. Through this case, we wanted to draw the attention of orthopaedic surgeons to the fact that the PAES can be asymptomatic. Its symptomatology can be triggered by post-traumatic instability of the knee. Its presence represents a risk of lesion of the popliteal artery during arthroscopic ACL reconstruction. Therefore, it is important to think about this disease if a calf pain occurring after a ligament injury of the knee.
